# Trends in Utilization and In-hospital Outcomes of Cardiac Surgery

**DOI:** 10.21470/1678-9741-1-2020-0603

**Published:** 2020

**Authors:** Javier de Miguel-Díez, Rodrigo Jiménez-García, Ana López-de-Andrés

**Affiliations:** 1 Respiratory Department, Hospital General Universitario Gregorio Marañón, Facultad de Medicina, Universidad Complutense de Madrid - UCM, Instituto de Investigación Sanitaria Gregorio Marañón - IiSGM, Madrid, Spain.; 2 Department of Public Health & Maternal and Child Health, Facultad de Medicina, Universidad Complutense de Madrid, Madrid, Spain.; 3 Preventive Medicine and Public Health Teaching and Research Unit, Facultad de Ciencias de la Salud, Universidad Rey Juan Carlos, Alcorcón, Madrid, Spain.

In recent decades, there have been improvements in medical and interventional treatments that have changed the profile of patients referred to cardiac surgery. They are usually older and complex, with more severe disease and greater comorbidity, which may explain the progressive increase in surgical risk. However, there is no doubt that technological advances have also occurred in cardiac surgery, which has contributed to the maintenance of morbidity and mortality rates despite a higher risk^[1,2]^.

National registries of interventions represent an excellent method to identify and evaluate the trends and changes that occur in cardiac surgery over time, as well as to know possible areas for improvement^[3,4]^. Through them we have been able to verify that, in the past decade, transcatheter aortic valve implantation (TAVI) has become a lifesaving, minimally invasive therapy for many patients with severe aortic valve stenosis. The safety and efficacy of TAVI were first established for patients who were inoperable or at high risk for surgical aortic valve replacement (SAVR). Subsequent studies of trends have revealed a shift towards a lower-risk population and significantly improved clinical outcomes, with an impressively improvement in survival after TAVI, mainly as a consequence of decreased 30-day mortality^[5]^.

However, conventional SAVR is a common procedure and is still growing, being increasingly offered to a greater number of patients that were not previously considered as candidates to this procedure. In addition, this intervention has excellent results, with a much lower than estimated mortality^[4]^. Recently, Kundi et al.^[6]^ have evaluated changes in volume, risk profile, and outcomes among elderly individuals undergoing SAVR after TAVI approval in the United States of America. They have reported that the TAVI introduction and dissemination in the early phase were associated with an expansion of SAVR to high-risk patients, without an observed reduction in the use of this surgical procedure. This expansion was associated with similar mortality among all SAVR patients, despite an increase in patient risk.

Patients with aortic stenosis are usually older and often present with multiple comorbidities. Among these is the chronic obstructive pulmonary disease (COPD). The presence and severity of COPD in patients undergoing SAVR have been associated with a higher incidence of postoperative pneumonia and higher inhospital and long-term mortality^[7-9]^. However, sophistication in surgical techniques and introduction of less invasive approaches have reduced morbidity and mortality related to surgical treatment of aortic valve disease^[10]^. In a recent study of a large national database, we did not find differences in in-hospital mortality (IHM) between patients with and without COPD beside the type of valve used, mechanical or bioprosthetic^[11]^. On the other hand, COPD patients with aortic stenosis undergoing a valvular replacement procedure through TAVI also do not have a worse prognosis compared to non-COPD patients during hospitalization^[12]^.

Type 2 diabetes mellitus (T2DM) is another disease that also is strongly related to in-hospital and short-term prognosis in patients with cardiovascular diseases in need of surgical or invasive interventions. It has been reported a great increase in the number of T2DM patients who underwent SAVR in Spain from 2001 to 2015^[13]^. However, we have found than T2DM patients with aortic stenosis undergoing a valvular replacement procedure through SAVR or TAVI did not have a worse prognosis compared to nondiabetic patients during hospitalization, showing lower IHM after multivariable adjustment^[14]^.

The number of isolated mitral repair surgery procedures has increased in recent years^[4]^. Patients undergoing this intervention commonly have ventricular dysfunction, atrial fibrillation, and heart failure. Although contemporary outcomes are excellent, earlier guideline-directed referral and increased frequency and quality of repair may further improve results of mitral valve operations^[15]^.

Among the factors associated with poor surgical mitral valve replacement (SMVR) outcomes are age, advanced valvular cardiomyopathy, and associated comorbidities. In fact, a substantial part of symptomatic patients with severe mitral valve disease are excluded from surgery by institutional heart teams for these reasons. In a large analysis of patients undergoing SMVR, we demonstrated that COPD was associated with a significantly higher IHM among patients who received a bioprosthetic valve, but this disease did not predict IHM among those receiving a mechanical valve^[16]^. T2DM patients have also IHM and major adverse cardiovascular and cerebrovascular events after mechanical and bioprosthetic SMVR, but these outcomes are not significantly different from those found among non-diabetic patients^[17]^.

Regarding to isolated valvular surgery on the tricuspid valve, previously unusual, it is becoming an increasingly frequent procedure. In addition, it is experiencing a clear decrease in mortality, probably related to an earlier referral to surgical treatment^[3,4]^.

Regarding coronary surgery, percutaneous coronary intervention (PCI) has been replacing coronary artery bypass graft (CABG) surgery for the treatment of heart diseases due to the relative benefits of the first over the second, including the noninvasiveness of the procedure and a faster patient recovery. This has led to an increase in PCI volume among most Organization for Economic Cooperation and Development - OECD member countries over the past 20 years. The total procedure volumes of PCI surpassed those of CABG in the 1990s, following the publication of studies outlining clinical outcomes of the relative benefits of PCI^[18,19]^.

COPD is an independent factor of poor outcome and mortality after coronary revascularization procedures. In a recent study, we analyzed trends over a period of 11 years in the hospitalization of subjects with and without COPD who underwent these interventions^[20]^. We found an increase in PCI procedure rates from 2001 to 2011 and a decline in hospital admissions for CABG in patients with COPD from 2003 to 2011.

People with diabetes represent an increasing proportion of coronary artery disease patients, many of whom are treated with revascularization procedures. Using the Spanish National Hospital Database, we found different trends from 2001 to 2011 in the hospitalization of subjects with and without T2DM who underwent coronary revascularization procedures^[21]^. We found an increase in PCI procedure rates over the study period and a decline in hospital admissions for CABG in patients with T2DM from 2003 to 2011.

Since there are continuous advances in CABG and PCI, surgical approaches to coronary artery diseases are increasingly refined, with the use of more arterial conduits, less invasive surgical approaches, and development of new types of stents for PCI. However, the debate continues on which approach is the best^[22]^.
